# Predicting the potential distribution of the endemic seabird *Pelecanus thagus* in the Humboldt Current Large Marine Ecosystem under different climate change scenarios

**DOI:** 10.7717/peerj.7642

**Published:** 2019-10-25

**Authors:** Jaime A. Cursach, Aldo Arriagada, Jaime R. Rau, Jaime Ojeda, Gustavo Bizama, Anderson Becerra

**Affiliations:** 1 Programa de Doctorado en Ciencias Mención Manejo y Conservación de Recursos Naturales, Universidad de Los Lagos, Puerto Montt, Chile; 2 Laboratorio de Limnología, Departamento de Acuicultura y Recursos Agroalimentarios, Universidad de Los Lagos, Osorno, Chile; 3 Laboratorio de Ecología, Departamento de Ciencias Biológicas & Biodiversidad, Universidad de Los Lagos, Osorno, Chile; 4 Laboratorio de Ecosistemas Marinos Antárticos y Subantárticos (LEMAS), Universidad de Magallanes, Punta Arenas, Chile; 5 Instituto de Ecología y Biodiversidad (IEB), Santiago, Chile; 6 School of Environmental Studies, University of Victoria, Victoria, British Colombia, Canada; 7 Facultad de Ciencias, Universidad de Chile, Santiago, Chile; 8 Programa de Master en Ciencias y Tecnología Espacial, Escuela de Ingeniería, Universidad del País Vasco, Bilbao, Spain

**Keywords:** Conservation, MaxEnt, South America

## Abstract

**Background:**

The effects of global climate change on species inhabiting marine ecosystems are of growing concern, especially for endemic species that are sensitive due to restricted distribution. One method employed for determining the effects of climate change on the distribution of these organisms is species distribution modeling.

**Methods:**

We generated a model to evaluate the potential geographic distribution and breeding distribution of the Peruvian pelican (*Pelecanus thagus*). Based on maximum entropy modeling (MaxEnt), we identified the environmental factors that currently affect its geographic distribution and breeding. Then we predicted its future distribution range under two climate change scenarios: moderate (rcp 2.6) and severe (rcp 8.5).

**Results:**

The mean daytime temperature range and marine primary productivity explain the current potential distribution and breeding of the pelican. Under the future climate change scenarios, the spatial distribution of the pelican is predicted to slightly change. While the breeding distribution of the pelican can benefit in the moderate scenario, it is predicted to decrease (near −20 %) in the severe scenario.

**Discussion:**

The current potential geographic distribution of the pelican is influenced to a large extent by thermal conditions and primary productivity. Under the moderate scenario, a slight increase in pelican breeding distribution is predicted. This increase in habitable area is explained by the climatic conditions in southern Chile, and those climatic conditions will likely be similar to the current conditions of the central coast of Chile. We predict that the coasts of southern Chile will constitute an important refuge for the conservation of the Peruvian pelican under future climate change scenarios.

## Introduction

Climate change is of increasing concern for seabirds because it negatively affects their conservation status and has become the third most important threat after exotic invasive species and incidental capture (Croxall et al., 2012). In turn, a great proportion of seabirds (e.g., of the Humboldt Current System) feed in a relatively narrow range of trophic levels, mainly on larger zooplankton, small pelagic fish, or squid (Quillfeldt & Masello, 2013). Most of the prey species consumed by seabirds are strongly affected by climate-induced changes on the productivity of phytoplankton, generating changes in both the abundance and fecundity of herbivorous zooplankton (small copepods and euphausiids). Consequently, carnivorous zooplankton and pelagic fish or squid are also affected (Crawford et al., 2008a; Crawford et al., 2008b; Wynn et al., 2007; Luczak et al., 2011). The dynamics of small pelagic fish have been studied intensively in the marine upwelling ecosystems such the Humboldt and Benguela currents, where the collapse of small populations of pelagic fish is often followed by severe decreases in the populations of seabirds (Crawford & Jahncke, 1999; Crawford et al., 2008a). Seabirds face multiple imminent threats (overfishing and incidental death, pollution, introduced species, habitat destruction, and human disturbance) that may seem more urgent than gradual climate change and its associated climate phenomena (Croxall et al., 2012; Quillfeldt & Masello, 2013). However, some of these threats are locally restricted, whereas the climate phenomena have the potential to alter an entire region and increase the cumulative pressures that affect many seabirds, especially endemic species (Quillfeldt & Masello, 2013; Jenouvrier et al., 2014).

The Peruvian pelican *Pelecanus thagus* (hereafter pelican) is a seabird endemic to the Humboldt Current Large Marine Ecosystem (HCLME) of South America. The pelican’s home range lies on the Pacific coast from southern Ecuador, through Peru down to southern Chile (BirdLife International, 2018). However, it breeding distribution is not continuous along the coast, but is very localized in certain coastal islands from Santa Clara Island (3°S) in southern Ecuador, to Mocha Island (38°S) in central Chile (Housse, 1945; Vinueza, Sornoza & Yáñez Muñoz, 2015). At the global level, the pelican is classified as near threatened (BirdLife International, 2018). In Peru, this species is considered endangered (MINAGRI, 2014). In Chile and Ecuador there is no classification concerning its conservation status, even though the Chilean coastline comprises more than 50% of pelican’s habitat range (Cursach et al., 2018). Between 2010 and 2015 the abundance of pelicans in Chile decreased significantly on the central coast, area encompasses the main breeding population (Cursach et al., 2018).

Predicting the response of biodiversity to climate change has developed into an active field of research (Bellard et al., 2012; Molinos et al., 2015; Pecl et al., 2017). Therefore, projections of species distribution models play an important role in alerting scientists and decision makers to assess the potential future risks of climate change (Pereira et al., 2010; Parmesan et al., 2011). Climate change may alter the suitability of habitat and contraction of the distribution range of several groups of marine and terrestrial organisms, including Southern Ocean seabirds (Marzloff et al., 2016; Krüger et al., 2018). The current study aims to generate models of the potential geographic distribution and breeding of the pelican, to identify the environmental factors that affect its current distribution, and to predict its future distribution range under two climate change scenarios (moderate and severe). Our hypothesis was that the spatial distribution and breeding distribution of the pelican will decrease and that the main cause of this will be climate change.

## Materials & Methods

### Species records

Pelican nesting and occurrence data were compiled from four main sources: the Neotropical Waterbird Census (https://lac.wetlands.org/), eBird (https://ebird.org/), the Global Biodiversity Information Facility (https://www.gbif.org/), and the literature. The geo-coordinates for each data point were referenced from the information in the literature or through the use of coordinates in Google Earth. We excluded duplicate or unclear locations and verified the accuracy of the data. We found a total of 4,818 georeferenced data points referring to pelican sightings (in resting place, nesting sites, coves, beaches, etc.), encompassing its entire geographic distribution from 2000 to 2015. Of these records, a subsampling was performed at a distance of 15 km (cell size), obtaining a total of 264 records, with which the modeling was performed. This subsampling were conducted in R, version 3.0.2 (R Development Core Team, 2013). The breeding distribution of the pelican was modeling with information for 34 nesting sites (Vinueza, Sornoza & Yáñez Muñoz, 2015; Zavalaga, 2015; Cursach et al., 2018).

### Environmental variables

The environmental variables used to characterize the current distribution (and breeding) of the pelican were selected based on climate and oceanography. The climate variables used in this study were downloaded from the EcoClimate database (http://www.ecoclimate.org) (Lima-Ribeiro et al., 2015). These variables were represented by maximum, minimum, and mean values of monthly, quarterly, and annual temperatures, and the precipitation values recorded between 1950 and 2000. These parameters provided a combination of means, extremes, and seasonal differences in variables known to influence the distribution of species (Root et al., 2003). With the species distribution modeling toolbox extension implemented in ArcGIS, all bioclimate variables that showed a correlation higher than 0.7 were eliminated (Brown, 2014). Finally, six climate variables were selected: annual mean temperature, mean daytime temperature range, isothermality, seasonality in temperature, annual precipitation, seasonality in precipitation. The oceanographic variables used were sea surface temperature (SST) and marine net primary productivity (mg C m^−2^ day^−1^), as they are considered the main descriptors of the spatial distribution of seabirds (Quillfeldt et al., 2015; Ingenloff, 2017). These variables were obtained from the National Oceanic and Atmospheric Administration (NOAA, http://www.ngdc.noaa.gov/). For the analyses, we used mean values per climate season for a period of nine years (2004 to 2013), totaling eight oceanographic variables. All environmental variables used in this study were interpolated by the kriging method, with a uniform resolution of 0.5° × 0.5° using the QGIS 3.2.0 software (Lima-Ribeiro et al., 2015; Varela, Lima-Ribeiro & Terribile, 2015).

To evaluate the effects of the different climate change scenarios on the spatial distribution of pelicans, we did not include the oceanographic variables. The future climate scenarios corresponded to those proposed by the Intergovernmental Panel on Climate Change (IPCC, 2014). These scenarios were obtained from the ecoClimate website (http://ecoclimate.org/), which contains climate models available for different temporal intervals. To do this, we used the model developed by the Community Climate System Model version 4 of the National Center for Atmospheric Research (Gent et al., 2011). This is due to the good results for the South-East Pacific (Larson, Pegion & Kirtman, 2018; Zheng et al., 2018).

The projections for the six preselected variables and the projected minimum and maximum trajectories of the concentrations of greenhouse gases were obtained. That is 2.6 and 8.5 rcp (representative concentration pathways), respectively. These values indicate increases in the heat absorbed by the planet Earth due to the concentration of greenhouse gases up to 2100, in each trajectory and expressed in watts per square meter. Thus, 2.6 rcp is the moderate projection for the scenario with the least climate change, whereas 8.5 rcp is a more pessimistic projection and represents a severe scenario with the greatest climate change (Taylor, Stouffer & Meehl, 2012).

### Modeling of the potential geographic distribution

The MaxEnt software (MaxEnt version 3.3.3k, http://www.cs.princeton.edu/ schapire/maxent/) has been frequently used for species distribution models under current and future climate scenarios (Phillips & Dudík, 2008). We used MaxEnt to model the geographic distribution of the pelican, including under two previously described climate change scenarios (Elith et al., 2006; Taylor, Stouffer & Meehl, 2012). The model was elaborated by MaxEnt auto-features (5,000 iterations). Logistic output was used for all analyses. The quality of the model was evaluated using the area under the curve (AUC) and the continuous Boyce index (Hirzel et al., 2006). AUC values can vary from 0 to 1, where a value greater than 0.9 is considered an indicator of “good” discrimination skills (Peterson et al., 2011). Values of the Boyce index vary between −1 and 1, where positive values indicate a model with predictions that are consistent with the distribution of observed presences in the evaluation dataset (Boyce, 2002). Both analyses were conducted in R using the “biomod2” package (R Development Core Team, 2013).

For each distribution model, a 30-fold cross-validation was used, with a data proportion of 25% for training and 75% for evaluation. The most important environmental variables were identified by estimating the relative contribution (%) to the model (Phillips, Anderson & Schapire, 2006). Jackknife test was used to evaluate the importance of the environmental variables for predictive modeling (Almalki et al., 2015).

## Results

### Model yield for potential distribution

**Figure 1:**
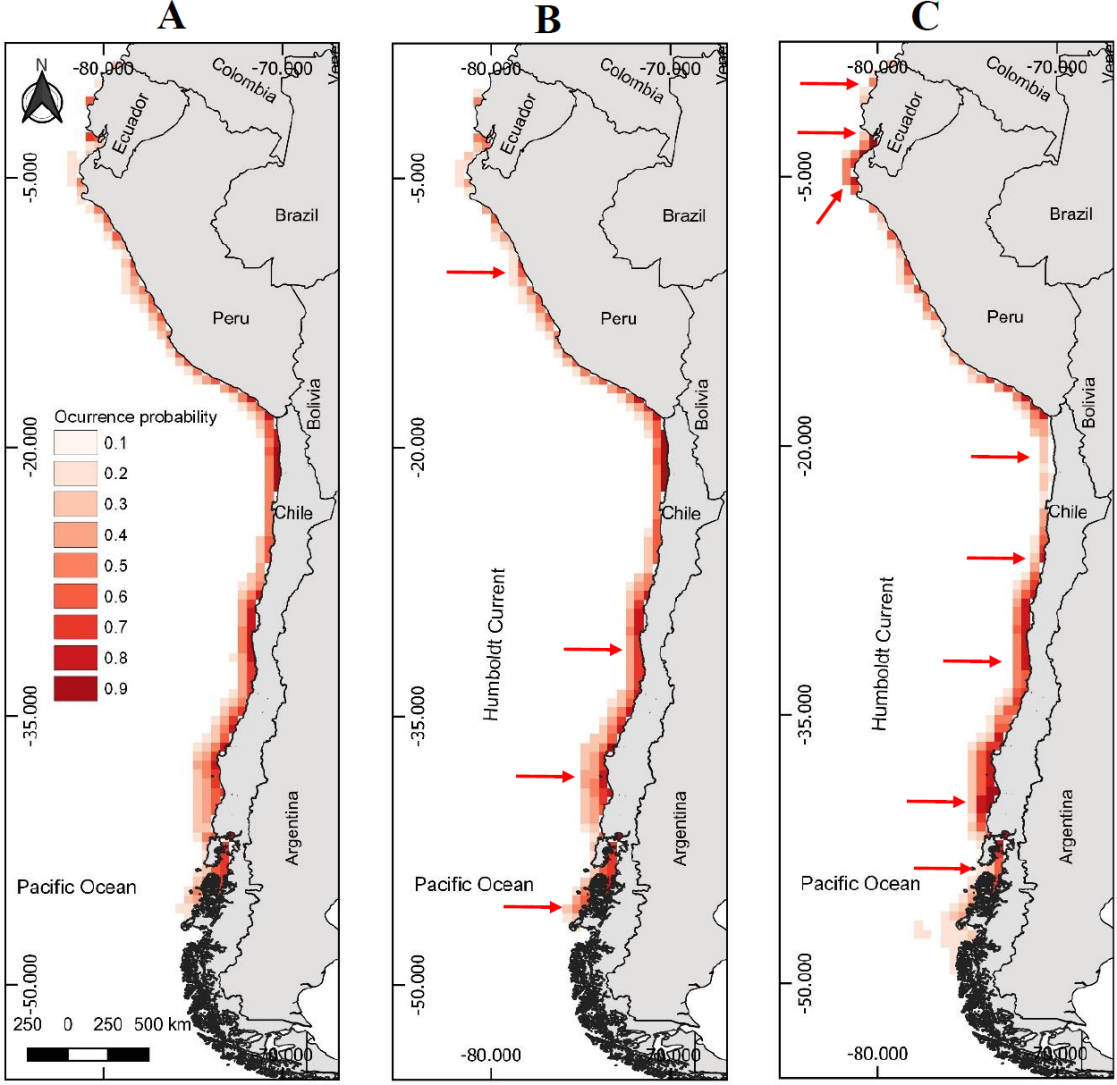
Models of potential geographic distribution of the Peruvian pelican (*P. thagus*) based on climatic variables and projected for 2010 according to two climate change scenarios. (A) Projection of current geographic distribution; (B) Projection at 2.6 rcp; (C) Projection at 8.5 rcp. The arrows show relative change to the current distribution.

**Figure 2:**
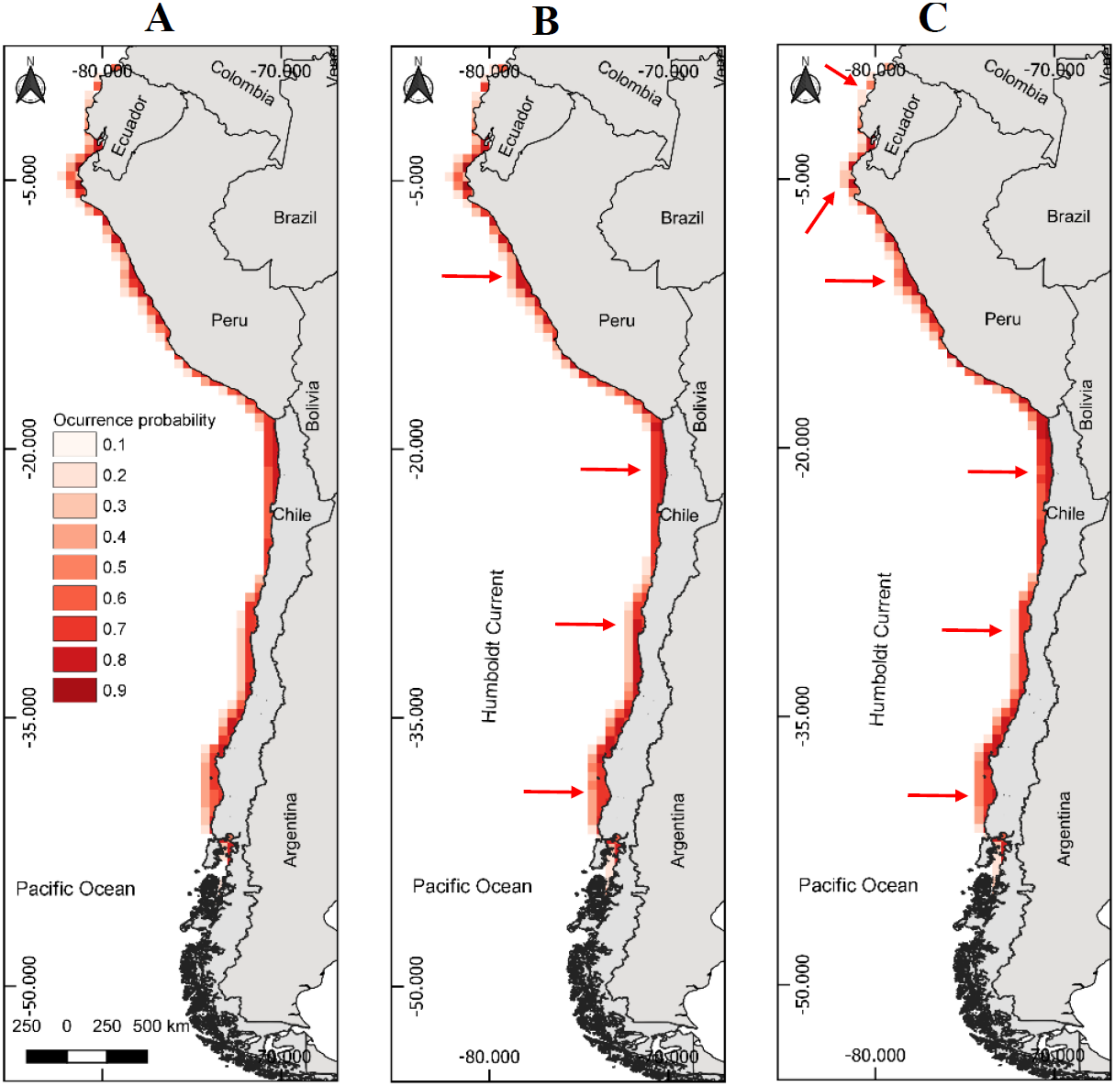
Models of potential reproductive distribution of the Peruvian pelican (*P. thagus*) based on climatic variables and projected for 2010 according to two climate change scenarios. (A) Projection of current reproductive distribution; (B) projection at 2.6 rcp; (C) projection at 8.5 rcp. The arrows show relative change to the current distribution.

The model of presence with the best fit showed a gain of 3.04 and a Boyce Index of 0.99. Also, an AUC_training_ of 0.98 and an AUC_evaluation_ of 0.98 and a standard deviation of 0.004. While the modeling of breeding distribution showed a gain of 2.24 and a Boyce Index of 0.98, with an AUC_training_ of 0.98 and an AUC_evaluation_ of 0.98 and a standard deviation of 0.003. The AUC values were relatively similar, so the models used are appropriate for predicting the presence and breeding distribution of the species. AUC_evaluation_ 0.98 indicates that the pelican has a wide geographic distribution and breeding in relation to the area corresponding to the environmental data. The model predicts that the potential geographic distribution of the pelican reaches an approximate surface area of 466,836 km^2^, latitudinally distributed from southern Ecuador (2°13′09″S) to southern Chile (46°59′07″S). Over this extensive marine–coastal surface, the probability of occurrence for this species varied between a 0.16 (minimum) and 0.84 (maximum) (Table 1). Areas with the highest probabilities of occurrence for the pelican are represented with intense red colors in Figs. 1A and 2A. These areas are mainly distributed from northern Peru to central Chile.

**Table 1:** Probability of occurrence ranges of the Peruvian Pelican (*Pelecanus thagus*) expressed in surface area.

**Potential geographic distribution**		**Potential reproductive distribution**	
**Probability of occurrence**	**Projected surface (km^2^)**	**Probability of occurrence**	**Projected surface (km^2^)**
0.16–0.25	174,841	0.1–0.2	103,148
0.25–0.33	82,153	0.2–0.3	49,407
0.33–0.42	40,498	0.3–0.4	63,245
0.42–0.50	59,119	0.4–0.5	31,296
0.50–0.59	43,793	0.5–0.6	28,232
0.59–0.67	36,910	0.6–0.7	110,200
0.67–0.76	18,950	0.7–0.8	88,326
0.76–0.84	10,572	0.8–0.9	0
**Total**	**466,836**	**Total**	**473,854**

### Importance of environmental variables

Among the six climatic variables and eight oceanographic variables, the mean daytime temperature range (Bio2) and the summer marine primary productivity, contributed the most to the current and potential distribution of the pelican (Table 2). These two factors explained 78.47% of the modeled distribution. The mean daytime temperature responded to the probability of the presence of the pelican, with a high probability of finding the species in areas where the mean daytime temperature ranges between 6 and 8 °C. In turn, the summer marine primary productivity also influenced the probability of the presence of the pelican, with a greater probability of finding the species during the summer season in areas with high primary productivity. The other factors such as, spring marine primary productivity, isothermality, and seasonality in temperature, contributed 9.24%, 3.23%, and 1.74%, respectively, to the modeled distribution. Therefore, thermal and primary productivity conditions are more important than other variables for mapping pelican distribution (Table 2).

**Table 2:** Contribution of environmental variables to the current potential distribution model of the Peruvian pelican (*Pelecanus thagus*).

**Variable**	**Contribution to the model (%)**	**Importance in permutation (%)**
Mean daytime temperature range	46.03	12.28
Summer marine primary productivity	32.44	1.26
Spring marine primary productivity	9.24	41.20
Isothermality	3.23	0.02
Seasonality in temperature	1.74	0.61
Sea surface temperature in winter	1.47	0.90
Sea surface temperature in spring	1.20	0.44
Sea surface temperature in summer	1.12	20.21
Seasonality of precipitation	1.10	11.54
Mean annual temperature	1.02	2.68
Annual precipitation	0.83	1.10
Fall marine primary productivity	0.41	3.35
Sea surface temperature in fall	0.11	4.34
Winter marine primary productivity	0	0

The modeling of breeding distribution showed that the mean daytime temperature range contributed with 91.5% to the model, while the summer marine primary productivity contributed with 8.5%.

### Potential geographic distribution of the pelican as a function of climate change

Based on the six climatic variables selected in the study, the model predicts that the projected pelican distribution currently attains an area of 596,753 km^2^ (Table 3). This area is larger than that initially projected (466,836 km^2^), where the oceanographic variables were integrated. Regarding the projections of climate change for 2100, under the moderate scenario of 2.6 rcp a slight decrease (−0.68%) in pelican spatial distribution is predicted (Table 3). Under the severe scenario of 8.5 rcp, a slight increase (4.51%) in pelican spatial distribution is predicted (Table 3).

**Table 3:** Probability of occurrence ranges of the Peruvian pelican (*Pelecanus thagus*) expressed in surface area, and those projected to 2100 under two climate change scenarios.

**Probability of occurrence**	**Projected surface (km^2^)**	**2.6 rcp scenario**		**8.5 rcp scenario**	
		**km**^2^	**Delta (km^2^)**	**km^2^**	**Delta (km^2^)**
0.089–0.17	111,147	115,583	4,436	160,747	49,600
0.17–0.26	109,380	92,394	−16,986	99,147	−10,233
0.26–0.35	80,529	77,101	−3,428	63,727	−16,802
0.35–0.44	58,849	62,374	3,525	53,352	−5,497
0.44–0.53	92,290	79,882	−12,408	62,344	−29,946
0.53–0.62	50,623	55,252	4,629	71,301	20,678
0.62–0.71	44,424	47,008	2,584	36,534	−7,890
0.71–0.80	32,837	35,903	3,066	55,481	22,644
0.80–0.89	16,674	27,161	10,487	21,059	4,385
**TOTAL**	**596,753**	**592,657**	**−4,096**	**623,692**	**26,939**

The projected habitable surface area under climate change of 2.6 rcp does not presents a major change with respect to the current geographic distribution of the pelican (Table 3). Under the severe scenario, the model predicts that the pelican habitable surface will vary depending on geographic area (Fig. 1). For example, in northern Chile its habitable surface would decrease, whereas in central and southern Chile it would increase over time (Fig. 2). The projected habitable surface area and the probabilities of occurrence for the pelican are spatially schematized in Fig. 2.

For the case of the modeling of breeding distribution, an area of 435,640 km^2^ is projected (Table 4). Regarding the projections of climate change for 2100, under the moderate scenario of 2.6 rcp an increase (8.77%) in pelican breeding distribution is predicted (Table 4). Under the severe scenario of 8.5 rcp, a decrease (−19.30%) in pelican breeding distribution is predicted (Table 4). Under the severe scenario, the model predicts a decrease of occurrence probability of nesting sites of the pelican in northern Ecuador and north-central Chile (Fig. 2).

**Table 4:** Probability of occurrence ranges of nesting sites of the Peruvian pelican (*Pelecanus thagus*) expressed in surface area, and those projected to 2100 under two climate change scenarios.

**Probability of occurrence**	**Projected surface (km^2^)**	**2.6 rcp scenario**		**8.5 rcp scenario**	
		**km**^2^	**Delta (km^2^)**	**km**^2^	**Delta (km^2^)**
0.1–0.2	75,037	103,148	28,111	88,676	13,639
0.2–0.3	61,766	49,407	−12,359	37,961	−23,805
0.3–0.4	51,566	63,245	11,679	55,694	4,128
0.4–0.5	28,432	31,296	2,864	41,435	13,003
0.5–0.6	61,167	28,232	−32,935	30,496	−30,671
0.6–0.7	102,422	110,200	7,778	86,676	−15,746
0.7–0.8	55,250	88,326	33,076	10,622	−44,628
**TOTAL**	**435,640**	**473,854**	**38,214**	**351,560**	**−84,080**

## Discussion

The potential geographic distribution of the pelican currently attains an approximate area of 466,836 km^2^, distributed latitudinally from southern Ecuador (2°13′09″S) to the Taitao Peninsula in southern Chile (46°59′07″S). While, the potential breeding distribution of the pelican currently attains an approximate area of 435,640 km^2^. The mean daytime temperature range and marine primary productivity explain the current potential distribution and breeding of the pelican, which is an endemic species closely associated with the oceanographic barriers of the Humboldt Current Ecosystem (Jeyasingham et al., 2013; Kennedy et al., 2013). In South America, the Humboldt Current encompasses the greater part of the Pacific coast. Despite the wide latitudinal gradient, the marine–coastal area exhibits a mean daytime temperature range between 4 °C and 8 °C. This is consistent with the highest probability of occurrence of the pelican (https://climatologia.meteochile.gob.cl/application/). In turn, marine productivity is the main predictor of biodiversity and especially of the presence of top predators such as seabirds (Wakefield, Phillips & Matthiopoulos, 2009). In the case of the pelican, there is an overlap between areas with high summer marine primary productivity and areas with nesting sites.

Under the future climate change scenarios, the spatial distribution of the pelican is predicted to slightly change. The pelican’s breeding distribution might be facilitated by the moderate scenario, increasing near 9%. However, under the severe scenario, the prediction decreased to near −20%. This trend is similar to other studies described for seabirds, whose breeding distribution will be reduced by climate change (Jenouvrier et al., 2014; Krüger et al., 2018). This increase in habitable area is explained by the climatic conditions in southern Chile, and those climatic conditions will likely be similar to the current conditions of the central coast of Chile (Falvey & Garreaud, 2009; Garreaud, 2011). Over the last decade, an increase in pelican abundance has been reported along the coast of southern Chile, with observations of large flocks following schools of pelagic fishes in the inner sea (Imberti, 2005; Häussermann, Forsterra & Plotnek, 2012; Cursach, Rau & Vilugrón, 2016; Cursach et al., 2018). In this area, there has even been one report of an unsuccessful attempt to nest (Cursach, Rau & Vilugrón, 2016). The occurrence of competitive interactions with other seabirds has also been observed with endemic species from Patagonia (Cursach, Rau & Vilugrón, 2016). In southern Chile, a group of pelicans was observed displacing nesting pairs of Imperial shag (*Phalacrocorax atriceps*), causing the abandonment of the nest (Cursach, Rau & Vilugrón, 2016).

The present study is one of only a few evaluations of the potential effects of climate change on seabirds on the Pacific coast of South America. To evaluate the different scenarios caused by climate change on the spatial distribution of the pelican, we did not include oceanographic variables. This is because the climatic variable “Mean daytime temperature range” was what largely explained the potential spatial distribution and breeding of the pelican. However, further studies are required to assess the effects of climate change on seabird populations, including oceanographic variables. In addition, it is important to recognize that the species spatial distribution models have methodological constraints, including operating based on climatic variables without integrating ecological interactions (Soberón, Osorio-Olvera & Peterson, 2017). The co-occurrence of fishing exploitation and El Niño events generates synergistic ecological effects that may push the pelican to critical levels of abundance (Passuni et al., 2016; Barbraud et al., 2018). In addition, the human disturbances on nesting sites are a key factor in the pelican population dynamics (Coker, 1919; Figueroa & Stucchi, 2012). Future modeling analyses should include field data about fishing, aquaculture, ENSO events, and human disturbances in nesting sites of the pelican.

In conclusion, the current potential geographic distribution of the pelican is influenced to a large extent by thermal conditions and primary productivity. Under the future climate change scenarios, the spatial distribution of the pelican is predicted to slightly change. The range of breeding distribution of the pelican will be decreased as the main cause of climate change. Under a moderate scenario, we predict that the coasts of southern Chile will constitute an important refuge for the conservation of the pelican. It is necessary that future investigations evaluate in detail the ecological interactions of the pelican and its population increase in southern Chile, considering the different dimensions of the local socio-ecological system.

##  Supplemental Information

10.7717/peerj.7642/supp-1 Supplemental Information 1 Occurrence data Georeferenced data points referring to pelican sightings (in resting place, nesting sites, coves, beaches, etc.), encompassing its entire geographic distribution from 2000 to 2015.

10.7717/peerj.7642/supp-2 Supplemental Information 2 Nesting data Confirmed information from 34 pelican nesting sites, in all their spatial distribution.

## Additional Information and Declarations

### Data Availability

The following information was supplied regarding data availability: Location data of pelican sightings are available as Supplemental Files.

## References

[ref-1] Almalki M, Alrashidi M, O’Connell M, Shobrak M, Szekely T (2015). Modelling the distribution of wetland birds on the Red Sea coast in the Kingdom of Saudi Arabia. Applied Ecology and Environmental Research.

[ref-2] Barbraud C, Bertrand A, Bouchón M, Chaigneau A, Delord K, Demarcq H, Gimenez O, Gutiérrez-Torero M, Gutiérrez D, Oliveros-Ramos R, Passuni G, Tremblay Y, Bertrand S (2018). Density dependence, prey accessibility and prey depletion by fisheries drive Peruvian seabird population dynamics. Ecography.

[ref-3] Bellard C, Bertelsmeier C, Leadley P, Thuiller W, Courchamp F (2012). Impacts of climate change on the future of biodiversity. Ecology Letters.

[ref-4] BirdLife International (2018). Species factsheet: *Pelecanus thagus*. http://www.birdlife.org.

[ref-5] Boyce MS (2002). Relating populations to habitats using resource selection functions. Ecological Modelling.

[ref-6] Brown JL (2014). SDMtoolbox: a python-based GIS toolkit for landscape genetic, biogeographic and species distribution model analyses. Methods in Ecology and Evolution.

[ref-7] Coker RE (1919). Habits and economic relations of the guano birds of Peru. Proceedings US National Museum.

[ref-8] Crawford RJM, Jahncke J (1999). Comparison of trends in abundance of guano-producing seabirds in Peru and southern Africa. South African Journal of Marine Science.

[ref-9] Crawford RJM, Sabarros PS, Fairweather T, Underhill LG, Wolfaardt AC (2008a). Implications for seabirds of a longterm change in the distribution of sardine: a South African experience. African Journal of Marine Science.

[ref-10] Crawford RJM, Tree AJ, Whittington PA, Visagie J, Upfold L, Roxburg KJ, Martin AP, Dyer BM (2008b). Recent distributional changes of seabirds in South Africa: is climate having an impact?. African Journal of Marine Science.

[ref-11] Croxall JP, Butchart SHM, Lascelles B, Stattersfield AJ, Sullivan B, Symes A, Taylor P (2012). Seabird conservation status, threats and priority actions: a global assessment. Bird Conservation International.

[ref-12] Cursach JA, Rau JR, Gelcich S, Rodríguez-Maulén J (2018). Situación poblacional del Pelícano Peruano (*Pelecanus thagus*) en Chile: prospección inicial. Ornitología Neotropical.

[ref-13] Cursach JA, Rau JR, Vilugrón J (2016). Presence of the Peruvian pelican (*Pelecanus thagus*) in seabird colonies of Chilean Patagonia. Marine Ornithology.

[ref-14] Elith J, Graham CH, Anderson RP, Dudk M, Ferrier S, Guisan A, Hijmans RJ, Huettmann F, Leathwick JR, Lehmann A, Li J, Lohmann LG, Loiselle BA, Manion G, Moritz C, Nakamura M, Nakazawa Y, Overton JM, Peterson AT, Phillips SJ, Richardson K, Scachetti-Pereira R, Schapire RE, Soberon J, Williams S, Wisz MS, Zimmermann NE (2006). Novel methods improve prediction of species’ distributions from occurrence data. Ecography.

[ref-15] Falvey M, Garreaud RD (2009). Regional cooling in a warming world: recent temperature trends in the southeast Pacific and along the west coast of subtropical South America (1979–2006). Journal of Geophysical Research.

[ref-16] Figueroa J, Stucchi M (2012). Isla Foca (Perú): registros de reproducción más septentrionales del pelícano (*Pelecanus thagus*) y del pilpilén negro (*Haematopus ater*). Boletín Chileno de Ornitología.

[ref-17] Garreaud R (2011). Cambio climático: bases físicas e impactos en Chile. Revista Tierra Adentro—INIA.

[ref-18] Gent PR, Danabasoglu G, Donner LJ, Holland MM, Hunke EC, Jayne SR, Lawrence DW, Neale RB, Rasch PJ, Vertenstein M, Worley PH (2011). The community climate system model version 4. Journal of Climate.

[ref-19] Häussermann V, Forsterra G, Plotnek E (2012). Sightings of marine mammals and birds in the Comau Fjord, Northern Patagonia, between 2003 and mid 2012 (Mammalia; Aves). Spixiana.

[ref-20] Hirzel AH, Lay GLe, Helfer V, Randin C, Guisan A (2006). Evaluating the ability of habitat suitability models to predict species presences. Ecological Modelling.

[ref-21] Housse R (1945). Las aves de Chile en su clasificación moderna, su vida y costumbres.

[ref-22] Imberti S (2005). Distribución otoñal de aves marinas y terrestres en los canales Chilenos. Anales del Instituto de la Patagonia.

[ref-23] Ingenloff K (2017). Biologically informed ecological niche models for an example pelagic, highly mobile species. European Journal of Ecology.

[ref-24] Intergovernamental Panel of Climate Change (2014). Climate change 2014: impacts, adaptation, and vulnerability. part a: global and sectoral aspects.

[ref-25] Jenouvrier S, Holland M, Stroeve J, Serreze M, Barbraud C, Weimerskirch H, Caswell H (2014). Projected continent-wide declines of the emperor penguin under climate change. Nature Climate Change.

[ref-26] Jeyasingham WS, Taylor SA, Zavalaga CB, Simeone A, Friesen VL (2013). Specialization to cold-water upwellings may facilitate gene flow in seabirds: new evidence from the Peruvian pelican *Pelecanus thagus* (Pelecaniformes: Pelecanidae). Journal of Avian Biology.

[ref-27] Kennedy M, Taylor SA, Nádvorník P, Spencer HG (2013). The phylogenetic relationships of the extant pelicans inferred from DNA sequence data. Molecular Phylogenetics and Evolution.

[ref-28] Krüger L, Ramos JA, Xavier JC, Gremillet D, González-Solís J, Petry MV, Phillips RA, Wanless RM, Paiva VH (2018). Projected distributions of Southern Ocean albatrosses, petrels and fisheries as a consequence of climatic change. Ecography.

[ref-29] Larson SM, Pegion KV, Kirtman BP (2018). The South Pacific Meridional Mode as a thermally driven source of ENSO amplitude modulation and uncertainty. Journal of Climate.

[ref-30] Lima-Ribeiro MS, Varela S, González-Hernández J, Oliveira G, Diniz-Filho JA, Terribile LC (2015). ecoClimate: a database of climate data from multiple models for past, present, and future for macroecologists and biogeographers. Biodiversity Informatics.

[ref-31] Luczak C, Beaugrand G, Jafré M, Lenoir S (2011). Climate change impact on Balearic shearwater through a trophic cascade. Biology Letters.

[ref-32] Marzloff MP, Melbourne-Thomas J, Hamon KG, Hoshino E, Jennings S, Van Putten IE, Pecl GT (2016). Modelling marine community responses to climate-driven species redistribution to guide monitoring and adaptive ecosystem-based management. Global Change Biology.

[ref-33] MINAGRI (2014). Decreto Supremo que aprueba la actualización de la lista de clasificación y categorización de las especies amenazadas de fauna silvestre legalmente protegidas (D.S. No 004–2014).

[ref-34] Molinos JG, Halpern BS, Schoeman DS, Brown CJ, Kiessling W, Moore PJ, Pandolfi JM, Poloczanska ES, Richardson AJ, Burrows MT (2015). Climate velocity and the future global redistribution of marine biodiversity. Nature Climate Change.

[ref-35] Parmesan C, Duarte CM, Poloczanska E, Richardson AJ, Singer MC (2011). Overstretching attribution. Nature Climate Change.

[ref-36] Passuni G, Barbraud C, Chaigneau A, Demarcq H, Ledesma J, Bertrand A, Castillo R, Perea A, Mori J, Viblanc V, Torres-Maita J, Bertrand S (2016). Seasonality in marine ecosystems: peruvian seabirds, anchovy, and oceanographic conditions. Ecology.

[ref-37] Pecl GT, Araujo MB, Bell J, Blanchard J, Bonebrake TC, Chen I, Clark TD, Colwell RK, Danielsen F, Evengard B, Falconi L, Ferrier S, Frusher S, García RA, Griffis RB, Hobday AJ, Janion-Scheepers C, Jarzyna MA, Jennings S, Lenoir J, Linnetved HI, Martin VY, McCormack PC, McDonald J, Mitchell NJ, Mustonen T, Pandolfi JM, Pettorelli N, Popova E, Sharon A. Robinson SA, Scheffers BR, Shaw JD, Sorte CJ, Strugnell JM, Sunday JM, Tuanmu M, Vergés A, Villanueva C, Wernberg T, Wapstra E, Williams SE (2017). Biodiversity redistribution under climate change: impacts on ecosystems and human well-being. Science.

[ref-38] Pereira HM, Leadley PW, Proenca V, Alkemade R, Scharlemann JPW, Fernandez-Manjarres JF, Araujo MB, Balvanera P, Biggs R, Cheung WWL, Chini L, Cooper HD, Gilman EL, Guenette S, Hurtt GC, Huntington HP, Mace GM, Oberdorff T, Revenga C, Rodrigues P, Scholes RJ, Sumaila UR, Walpole M (2010). Scenarios for global biodiversity in the 21st century. Science.

[ref-39] Peterson AT, Soberón J, Pearson RG, Anderson RP, Martinez-Meyer E, Nakamura M, Araujo MB (2011). Ecological niches and geographic distributions.

[ref-40] Phillips SJ, Anderson RP, Schapire RE (2006). Maximum entropy modeling of species geographic distributions. Ecological Modelling.

[ref-41] Phillips S, Dudík M (2008). Modeling of species distributions with Maxent: new extensions and a comprehensive evaluation. Ecography.

[ref-42] Quillfeldt P, Cherel Y, Delord K, Weimerkirch H (2015). Cool, cold or colder? Spatial segregation of prions and blue petrels is explained by differences in preferred sea surface temperatures. Biology Letters.

[ref-43] Quillfeldt P, Masello JF (2013). Impacts of climate variation and potential effects of climate change on South American seabirds—a review. Marine Biology Research.

[ref-44] R Development Core Team (2013). R: a language and environment for statistical computing.

[ref-45] Root TL, Price JT, Hall KR, Schneider SH, Rosenzweig C, Pounds JA (2003). Fingerprints of global warming on wild animals and plants. Nature.

[ref-46] Soberón J, Osorio-Olvera L, Peterson T (2017). Diferencias conceptuales entre modelación de nichos y modelación de áreas de distribución. Revista Mexicana de Biodiversidad.

[ref-47] Taylor KE, Stouffer RJ, Meehl GA (2012). An overview of CMIP5 and the experiment design. Bulletin of the American Meteorological Society.

[ref-48] Varela S, Lima-Ribeiro MS, Terribile LC (2015). A short guide to the climate variables of the last glacial maximum for biogeographers. PLOS ONE.

[ref-49] Vinueza GS, Sornoza F, Yáñez Muñoz MH (2015). Primer registro de anidación del pelícano peruano *Pelecanus thagus* (Pelecaniformes: Pelecanidae) en Ecuador. Avances en Ciencias e Ingenierías.

[ref-50] Wakefield ED, Phillips RA, Matthiopoulos J (2009). Quantifying habitat use and preferences of pelagic seabirds using individual movement data: a review. Marine Ecology Progress Series.

[ref-51] Wynn RB, Josey SA, Martin AP, Johns DG, Yésou P (2007). Climate-driven range expansion of a critically endangered top predator in northeast Atlantic waters. Biology Letters.

[ref-52] Zavalaga CB (2015). Índices para el inicio y cierre de las campañas de extracción de guano en la RNSIIPG (Especial atención a los aspectos reproductivos de las tres especies de aves guaneras y considerando como caso de estudio a la Isla Guañape Sur).

[ref-53] Zheng J, Wang F, Alexander MA, Wang M (2018). Impact of South Pacific subtropical dipole mode on the equatorial Pacific. Journal of Climate.

